# Can flash glucose monitoring improve glucose management for Aboriginal and Torres Strait Islander peoples with type 2 diabetes? A protocol for a randomised controlled trial

**DOI:** 10.1186/s13063-024-08267-7

**Published:** 2024-07-19

**Authors:** Mariam Hachem, Tracey Hearn, Ray Kelly, Audrey Eer, Belinda Moore, Christine Sommerville, Sharon Atkinson-Briggs, Stephen Twigg, Meagan Freund, David O’Neal, David Story, Alex Brown, Anna McLean, Ashim Sinha, John Furler, Richard O’Brien, An Tran-Duy, Philip Clarke, Sabine Braat, Digsu N. Koye, Sandra Eades, Luke Burchill, Elif Ekinci

**Affiliations:** 1 Department of Medicine, Austin Health, Faculty of Medicine, Dentistry and Health Sciences, University of Melbourne, Melbourne, Victoria Australia; 2 Endocrinology, Austin Health, Melbourne, Victoria Australia; 3 Australian Centre for Accelerating Diabetes Innovations (ACADI), Melbourne Medical School, Faculty of Medicine, Dentistry and Health Sciences, University of Melbourne, Melbourne, Victoria Australia; 4 Medical Clinic, Rumbalara Aboriginal Cooperative, Mooroopna, Victoria Australia; 5 Implementation Science Research Group, University of Melbourne, Melbourne, Victoria Australia; 6 School of Medicine and Public Health, College of Health, Medicine and Wellbeing, Health Behaviour Research Collaborative, University of Newcastle, Newcastle, New South Wales Australia; 7 Department of Medicine, FacultyofMedicine,DentistryandHealth Sciences, University of Melbourne, Melbourne, Victoria Australia; 8 Endocrinology, St Vincent’s Hospital Melbourne, Melbourne, Victoria Australia; 9 Critical Care, FacultyofMedicine,DentistryandHealthSciences, University of Melbourne, Melbourne, Victoria Australia; 10 Indigenous Genomics, Telethon Kids Institute, Adelaide, South Australia Australia; 11 Indigenous Genomics, The Australian National University, Canberra, Australian Capital Territory Australia; 12 Department of Endocrinology and Diabetes, Cairns Hospital, Cairns, Queensland Australia; 13 Department of General Practice, University of Melbourne, Melbourne, Victoria Australia; 14 Centre for Health Policy, Melbourne School of Population and Global Health, University of Melbourne, Melbourne, Victoria Australia; 15 Methods and Implementation Support for Clinical and Health research (MISCH) Hub, Faculty of Medicine, Dentistry and Health Sciences, University of Melbourne, Melbourne, Victoria Australia; 16 Health Economics Research Centre, University of Oxford, Oxford, United Kingdom; 17 Centre for Epidemiology and BiostatisticsMelbourne School of Population and Global HealthFaculty of Medicine, Dentistry and Health Sciences, University of Melbourne, Melbourne, Victoria Australia; 18 Cardiovascular Medicine, Rochester, Minnesota United States of America

**Keywords:** Type 2 diabetes, Aboriginal and torres Strait Islander peoples, Indigenous Australians, First Nations, Continuous glucose monitoring, Flash Glucose Monitoring, HbA1c, Randomised controlled trial, Freestyle Libre 2

## Abstract

**Background:**

Aboriginal and Torres Strait Islander peoples are disproportionately impacted by type 2 diabetes. Continuous glucose monitoring (CGM) technology (such as Abbott Freestyle Libre 2, previously referred to as Flash Glucose Monitoring) offers real-time glucose monitoring that is convenient and easy to use compared to self-monitoring of blood glucose (SMBG). However, this technology’s use is neither widespread nor subsidised for Aboriginal and Torres Strait Islander peoples with type 2 diabetes. Building on existing collaborations with a national network of Aboriginal and Torres Strait Islander communities, this randomised controlled trial aims to assess the effect of CGM compared to SMBG on (i) haemoglobin A1c (HbA1c), (ii) achieving blood glucose targets, (iii) reducing hypoglycaemic episodes and (iv) cost-effective healthcare in an Aboriginal and Torres Strait Islander people health setting.

**Methods:**

This is a non-masked, parallel-group, two-arm, individually randomised, controlled trial (ACTRN12621000753853). Aboriginal and Torres Strait Islander adults with type 2 diabetes on injectable therapy and HbA1c ≥ 7.5% (*n* = 350) will be randomised (1:1) to CGM or SMBG for 6 months. The primary outcome is change in HbA1c level from baseline to 6 months. Secondary outcomes include (i) CGM-derived metrics, (ii) frequency of hypoglycaemic episodes, (iii) health-related quality of life and (iv) incremental cost per quality-adjusted life year gained associated with the CGM compared to SMBG. Clinical trial sites include Aboriginal Community Controlled Organisations, Aboriginal Medical Services, primary care centres and tertiary hospitals across urban, rural, regional and remote Australia.

**Discussion:**

The trial will assess the effect of CGM compared to SMBG on HbA1c for Aboriginal and Torres Strait Islander people with type 2 diabetes in Australia. This trial could have long-term benefits in improving diabetes management and providing evidence for funding of CGM in this population.

**Trial registration:**

Australian and New Zealand Clinical Trials Registry ACTRN12621000753853. Registered on 15th June 2021.

## Administrative information

Note: the numbers in curly brackets in this protocol refer to SPIRIT checklist item numbers. The order of the items has been modified to group similar items (see http://www.equator-network.org/reporting-guidelines/spirit-2013-statement-defining-standard-protocol-items-for-clinical-trials/).

**Table:** 

Title {1}	Can Flash Glucose Monitoring improve glucose management for Aboriginal and Torres Strait Islander people with type 2 diabetes? A protocol for a randomised controlled trial
Trial registration {2a} and {2b}	Australian and New Zealand Clinical Trials Registry (ACTRN12621000753853). Registration date: 15th June 2021
Protocol version {3}	Version 1.4, dated 1st June 2023
Funding {4}	The Australian National Health and Medical Research Council (NHMRC) Clinical Trials and Cohort Studies Grant 2020: GNT1182464
Author details {5a}	Mariam Hachem^1,2,3^, Tracey Hearn^1,3,4^, Ray Kelly^1,3^, Audrey Eer^1,2^, Belinda Moore^3,5^, Christine Sommerville^1,3^, Sharon Atkinson-Briggs^4^, Stephen Twigg^3^, Meagan Freund^6^, David O’Neal^3,7,8^, David Story^9^, Alex Brown^10,11^, Anna McLean^12^, Ashim Sinha^12^, John Furler^13^, Richard O’Brien^2^, An Tran- Duy^3,14,17^, Philip Clarke^3,14,15^, Sabine Braat^16,17^, Digsu N. Koye^3,16,17^, Sandra Eades^14^, Luke Burchill^18^, Elif Ekinci^1,2,3^ ^1^Department of Medicine—Austin Health, Faculty of Medicine, Dentistry and Health Sciences, University of Melbourne, Melbourne, VIC, Australia ^2^Endocrinology, Austin Health, Melbourne, VIC, Australia ^3^Australian Centre for Accelerating Diabetes Innovations (ACADI), Melbourne Medical School, Faculty of Medicine, Dentistry and Health Sciences, University of Melbourne, Melbourne, VIC, Australia ^4^Medical Clinic, Rumbalara Aboriginal Cooperative, Melbourne, VIC, Australia ^5^Implementation Science Research Group, University of Melbourne, Melbourne, VIC, Australia ^6^Health Behaviour Research Collaborative, School of Medicine and Public Health, College of Health, Medicine and Wellbeing, University of Newcastle, Newcastle, NSW, Australia ^7^Department of Medicine, Faculty of Medicine, Dentistry and Health Sciences, University of Melbourne, Melbourne, VIC, Australia ^8^Endocrinology, St Vincent’s Hospital Melbourne, Melbourne, VIC, Australia ^9^Critical Care, Faculty of Medicine, Dentistry and Health Sciences, University of Melbourne, Melbourne, VIC, Australia ^10^Indigenous Genomics, Telethon Kids Institute, Adelaide, SA, Australia ^11^Indigenous Genomics, The Australian National University, Canberra, ACT, Australia ^12^Department of Endocrinology and Diabetes, Cairns Hospital, Cairns, QLD, Australia ^13^Department of General Practice, University of Melbourne, Melbourne, VIC, Australia ^14^Centre for Health Policy, Melbourne School of Population and Global Health, The University of Melbourne, Melbourne, VIC, Australia ^15^Health Economics Research Centre, University of Oxford, Oxford, UK ^16^Centre for Epidemiology and Biostatistics, Melbourne School of Population and Global Health, Faculty of Medicine, Dentistry and Health Sciences, University of Melbourne, Melbourne, VIC, Australia ^17^Methods and Implementation Support for Clinical and Health research (MISCH) Hub, Faculty of Medicine, Dentistry and Health Sciences, University of Melbourne, Melbourne, VIC, Australia ^18^Cardiovascular Medicine, Mayo Clinic, Rochester, Minnesota, USA
Name and contact information for the trial sponsor {5b}	Victoria McMorran, Office of Research, Ethics and Integrity Research, Innovation and Commercialisation, Chief Operating Officer Portfolio, The University of Melbourne, Victoria 3010 Australia clinicaltrials-governance@unimelb.edu.au
Role of sponsor {5c}	The study sponsor and funders were not involved in the study design, data collection, management, analysis, or interpretation of the data; writing of the manuscript; nor the decision to submit for publication.

## Introduction

### Background and rationale {6a}

Diabetes is the fastest growing chronic condition globally, with type 2 diabetes considered a widespread epidemic of significant proportions [1, 2]. In Australia, over 13% of the Aboriginal and Torres Strait Islander population are affected by diabetes compared to 4.5% of the total Australian population [3, 4]. Recognised as a priority area, The National Aboriginal and Torres Strait Islander Health Plan 2021–2031 calls for direct action to reduce the burden diabetes and its complications (cardiovascular disease, eye disease, renal disease) [5]. The Australian Institute for Health and Welfare 2023 reports diabetes as the 5th leading cause of death for Aboriginal and Torres Strait Islander peoples between 2015 and 2019 [3]. Furthermore, the overall rate of diabetes-related admissions for Aboriginal and Torres Strait Islander peoples is more than five times that of non-Aboriginal and Torres Strait Islander peoples matched for age, sex and residence [6]. For Aboriginal and Torres Strait Islander people, diabetes and its management impose significant social, cultural and financial costs that impact functioning at the individual and community level.

Many large, international, multi-centre diabetes trials have demonstrated the effectiveness of medications (such as injectable insulin, oral hypoglycaemic agents, injectable glucagon-like peptide-1-receptor agonists) and technologies in reducing blood glucose and micro- and macro-vascular diabetes complications [7–13]. Yet many Australians with diabetes do not meet recommended target levels for blood glucose (3.9–10 mmol/L), particularly in remote Aboriginal and Torres Strait Islander communities [14]. For every 1% increase in HbA1c, the risk of serious and costly medical complications increases by 21% [15]. To improve outcomes for Aboriginal and Torres Strait Islander peoples with type 2 diabetes, addressing barriers such as disparities in timely diabetes prevention, management and treatment including access to diabetes technologies, service provision and culturally safe and appropriate care are required [5].

Diabetes technology such as continuous glucose monitoring (CGM) has revolutionised diabetes management, replacing the need for self-monitoring of blood glucose (SMBG) [16]. CGM is recognised by peak diabetes bodies for improving glycaemic management, particularly for people with type 1 diabetes [17–19] with growing evidence of its use for type 2 diabetes. The IMpact of flash glucose Monitoring in pEople with type 2 Diabetes Inadequately controlled with non-insulin Antihyperglycaemic ThErapy (IMMEDIATE) study demonstrated that CGM usage improved glycated haemoglobin (HbA1c) reduction of 0.3% (3 mmol/mol), increased time in range by 9.9% (or 2.4 h/day) and decreased time above range by 8.1% (or 1.9 h/day) for people with type 2 diabetes using non-insulin therapies [20]. For people aged < 65 years with type 2 diabetes and on intensive insulin therapy, HbA1c reduction was significantly greater in those using CGM for 6 months compared to SMBG, with less time spent in hypoglycaemia, and improved quality of life [21]. CGM provides clear, comprehensive and sufficient glucose level data with minimal user inconvenience and improves clinical care [21]. Real-time CGM has also been reported to foster self-monitoring and increased physical activity time for people with type 2 diabetes [22]. Other studies have also demonstrated the reductions in HbA1c using data from CGM leads to cost-savings for health systems [23–25]. Cost and effectiveness are critical issues, especially for Aboriginal and Torres Strait communities with type 2 diabetes who are at high risk of serious complications [26]. However, to date, no trials have demonstrated the benefits of CGM in an Aboriginal and Torres Strait Islander health context.

This trial will examine one such device, the FreeStyle® Libre™ 2 CGM system (Abbott Diabetes Care, Alameda, CA) (ARTG ID 358292) [27] for Aboriginal and Torres Strait Islander people with type 2 diabetes living in metropolitan, rural, regional and remote Australia.

### Objectives {7}

The primary aim of this trial is to assess the effects of using CGM on HbA1c compared to SMBG. Secondary aims are to assess effects of using CGM compared to SMBG on (i) CGM-derived metrics, (ii) frequency of hypoglycaemic episodes, (iii) health-related quality of life and (iv) incremental cost per quality-adjusted life year gained associated with the CGM compared to SMBG*.*

### Trial design {8}

**Fig. 1:**
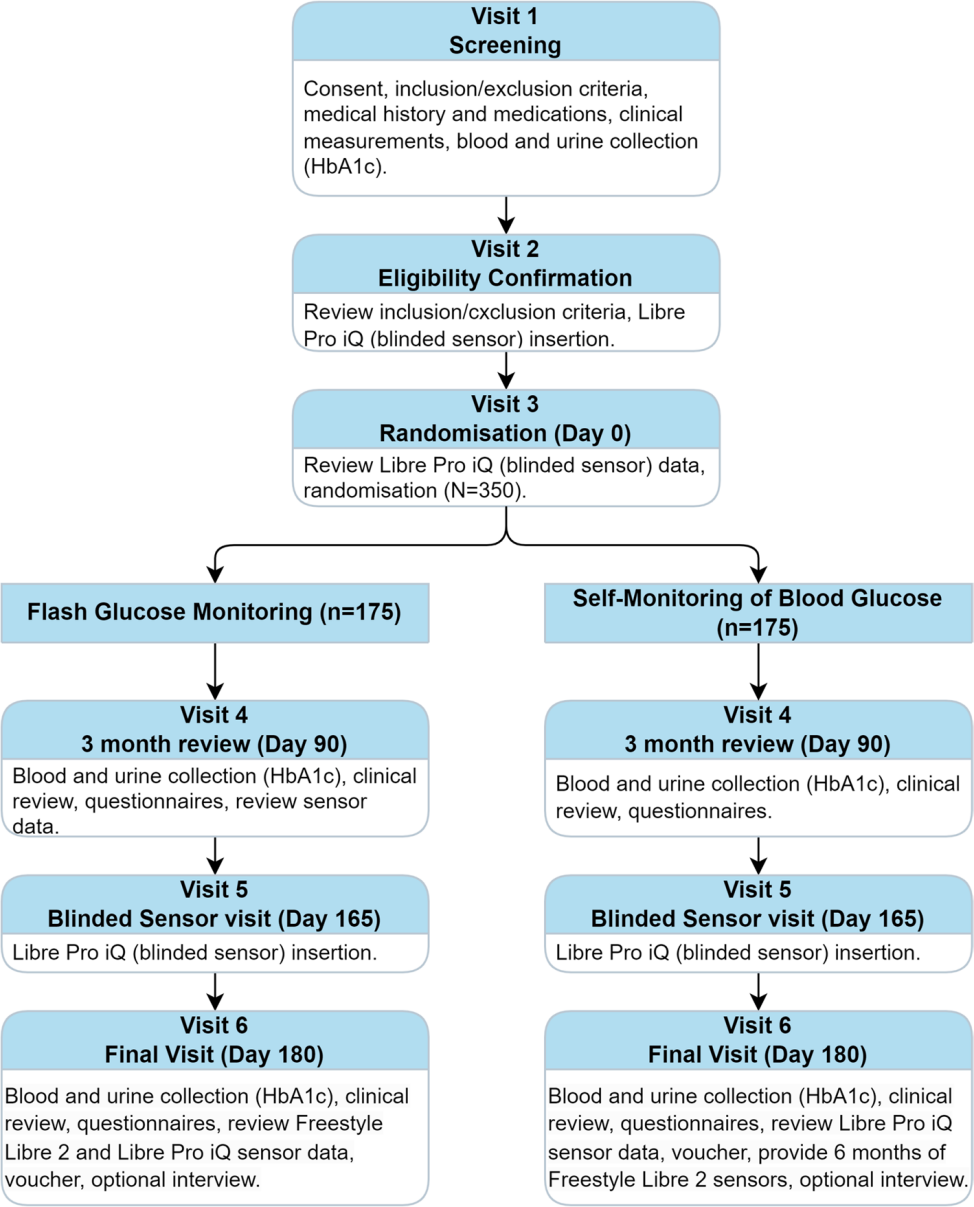
Trial diagram

The FlashGM Study is multi-centre, non-masked, parallel-group, two-arm, individually randomised (1:1), controlled superiority trial for Aboriginal and Torres Strait Islander peoples living in Australia with type 2 diabetes on injectable diabetes therapies, comparing CGM to standard care (SMBG) for 6 months (study flow chart outlined in Fig. 1). The trial methods were piloted in a feasibility study with 40 Aboriginal and Torres Strait Islander people with type 2 diabetes [28]. CGM technology and the study was well received by participants; however, the design was not statistically powered to draw conclusions. This multi-centre, national, randomised controlled trial is powered to detect a change in HbA1c from baseline to 6 months using CGM for Aboriginal and Torres Strait Islander people with type 2 diabetes.

## Methods: participants, interventions and outcomes

### Study setting {9}

The trial is recruiting in clinical trial sites in metropolitan, regional, rural and remote centres across Australia. Trial sites include Aboriginal Community Controlled Health Organisations, Aboriginal Medical Services and primary care centres and tertiary hospitals with diabetes services. Recruiting sites must demonstrate community engagement, presence of and/or access to diabetes education services, and healthcare workers/clinicians who can be responsive to CGM technology data and provide care for participants on the trial. Trial sites are chosen on the basis that community needs are prioritised over all other factors, with local priorities and contextual factors being of utmost importance (i.e. cultural, community and family responsibilities, workforce capacity and extenuating circumstances such as natural disasters). Due to extenuating circumstances where participants are unable to physically attend clinic visits due to remoteness, change of location, COVID-19 illness or other circumstances, telehealth visits may be conducted.

### Eligibility criteria {10}

Persons meeting all the following criteria and none of the exclusion criteria will be eligible for enrolment:

1. Identify as Aboriginal and Torres Strait Islander person
2. Confirmed diagnosis of type 2 diabetes
3. Age ≥ 18 years
4. HbA1c ≥ 7.5% (58 mmol/mol) from either a recent venous blood test (within 2 weeks of screening date) or blood sample taken at screening determined through laboratory pathology testing
5. Medicated with the same type of injectable therapies ± oral hypoglycaemic agents, for at least 6 weeks prior to screening:
  i. Insulin alone or with oral hypoglycaemic agents
  ii. Glucagon-like peptide 1 (GLP-1) analogues and subclasses alone, or with oral hypoglycaemic agents
  iii. GLP-1 analogues and subclasses, and insulin alone, or with oral hypoglycaemic agents

### Exclusion criteria

Persons meeting any of the following criteria will not be eligible:

1. Taking high doses of vitamin C supplements (more than 500 mg per day)
2. Active malignancy requiring chemotherapy
3. Known allergy to medical grade adhesives
4. On varying doses of corticosteroid therapy
5. Using amphetamines, anabolic or weight-reducing therapies
6. Pregnancy or actively planning pregnancy as HbA1c is not reliable during pregnancy
7. Estimated glomerular filtration rate < 15 mL/min/1.732 or erythropoiesis stimulating agents or end-stage kidney disease
8. Haemoglobinopathies
9. Active illicit drug use or heavy alcohol use
10. No informed consent

### Who will take informed consent? {26a}

Participants are collaboratively identified by study and clinical staff at each trial site. Study staff include credentialled diabetes nurse educators, Aboriginal Health workers, Aboriginal Health practitioners, nurse practitioners, general practitioners, endocrinologists and allied health professionals. Informed consent is undertaken by study staff who have completed ICH-GCP training (Good Clinical Practice). Discussions with potential participants are undertaken using the informed consent video and the participant informed consent form (PICF) provided. The informed consent video has been developed to facilitate consenting in a culturally appropriate way for the trial [29]. The video includes brief information about what the study is about, what is involved at each study visit and provides an opportunity for participants to ask questions about the study. The video has been developed by key Aboriginal study members (Tracey Hearn, Luke Burchill) alongside the study artist (Bernard Kelly-Edwards). Aboriginal Health workers and Aboriginal Health practitioners who are part of the clinical service can provide translation to the PICF for those who require translation. Signatures will be provided by the participant undertaking the trial, the study staff member facilitating the consent process and a witness, if required. Upon signing, consent will be marked complete for the participant.

### Additional consent provisions for collection and use of participant data and biological specimens {26b}

This trial does not involve storage of biological specimens. Biological samples are only collected and analysed for routine pathology at visit 1, visit 4 and visit 6 at an accredited pathology provider.

### Interventions

#### Explanation for the choice of comparators {6b}

Participants randomised to standard care (SMBG) will self-monitor blood glucose levels (BGL) by finger-pricking using their usual glucose metres. They will be instructed to follow their usual diabetes care procedures. This may include advice for twice daily those on medications that do not include insulin where appropriate and up to four times per day for those using insulin. Furthermore, participants will be asked to check their BGL when experiencing symptoms of hypoglycaemia or hyperglycaemia. Data will be collected on the number of times participants check their BGL in both arms.

#### Intervention description {11a}

##### Freestyle Libre 2

The study intervention is the Freestyle Libre 2, a CGM device (Abbott Diabetes Care, Australia). The device is worn on the upper arm for 14 days at a time. It uses wired enzyme technology, coated with glucose oxidase, to measure interstitial BGL. The device has a Bluetooth-enabled sensor that communicates with an Abbott reader and/or the Freestyle LibreLink application (on an Android or iOS smartphone), displaying current and historical glucose data in 1-min increments. Participants allocated to receiving CGM will be given a choice (at randomisation) between the reader and the smartphone application and will be asked to bring their device to every study visit. At commencement, the optional hypoglycaemic alarm is standardised by study staff to detect low glucose levels < 4.0 mmol/L during the trial. While it is recommended that the hyperglycaemic alarm is set at > 14.0 mmol/L, participants and clinicians have the flexibility to adjust this based on clinical judgement, participant experience and preference. Participants are advised to scan with purpose at least 10 times per day, to contact their treating clinician if they are concerned about glucose levels or the Freestyle Libre 2 system, and to do a finger prick test if their glucose readings and alerts do not match symptoms or expectations. However, their diabetes team will support them with this as this interaction also provides an opportunity to provide tailored diabetes education. Sensors, the reader and the smartphone application are supplied at no cost to the participant and instructions will be given to the participant at the first fitting [30].

Sensor data for participants in the intervention group will be made available to the participants and their clinicians for personalised diabetes management. The treating clinicians will receive training regarding use of the CGM system, how to access sensor data and how to analyse glycaemic profile results, known as an ambulatory glucose profile (AGP). The AGP is an internationally agreed standard for summarising and interpreting glycaemic data in a visual format [31]. This allows health professionals and people with diabetes to identify patterns and trends in daily glucose trends, including hypoglycaemic and hyperglycaemic episodes [31]. The AGP is useful in targeting changes to aspects of lifestyle that can optimise glycaemia [31]. Clinicians will view the AGP report for each participant as part of the trial. Latest guidelines for interpreting AGP reports will be used.

##### Blinded sensor (Freestyle LibrePro iQ, Abbott Pty Ltd)

The Freestyle LibrePro iQ is a blinded sensor that will be worn by all participants between the eligibility confirmation visit (visit 2) and randomisation visit (visit 3) for a period of 2 weeks only. At visit 2, the study staff member will explain the importance of wearing a blinded sensor and instruct the participant to continue to monitor their BGL as per standard care procedures. This is important as the data collected from the blinded sensors will provide the secondary outcomes at the end of the trial from both the intervention and control arm. Similarly, all participants will wear the Freestyle LibrePro iQ blinded sensor for 2 weeks between the blinded sensor insertion visit (visit 5) and the final visit (visit 6). The participant will be instructed to leave the sensor on for 2 weeks. Data from the blinded sensors inform several secondary outcomes for the study; therefore, the importance of wearing the blinded sensor and continuing to monitor their blood glucose is emphasised when the sensor is fitted. At visit 3 (randomisation) and visit 6 (final visit), the blinded sensor will be scanned by a health professional to retrieve the blood glucose data collected during the previous 2-week period. Upon confirmation that the data has been captured and downloaded onto the Freestyle Libreview software, the study staff member will remove the Freestyle LibrePro sensor from the participant. A 14-day AGP is provided for and explained to each participant.

### Criteria for discontinuing or modifying allocated interventions {11b}

There will be no special criteria for discontinuing or modifying allocated interventions.

### Strategies to improve adherence to interventions {11c}

All participants will receive diabetes education at randomisation to collaboratively discuss their diabetes management goals. Diabetes education topics relevant to the FlashGM Study to be revisited at each scheduled study visit include hypoglycaemia and hyperglycaemia experiences; sensor glucose scanning times and targets; self-monitoring blood glucose checking times and targets; oral medication tolerance and adherence; insulin, injection technique; GLP-1 analogue injection technique; nutrition, physical activity and weight management; sick day management and complication screening. General diabetes education is to be delivered to all participants from a strengths-based perspective allowing the health professional to meet the participant with they are at so personalised diabetes education, in a manner that meets the need of the individual’s health literacy status. Additionally, participant incentives to complete the trial include a $50 voucher and participant study t-shirt, provision of post-trial compassionate access to CGM if randomised to standard care, and compassionate access to GLP-1 injectables due to an ongoing global shortage (2023 to present) [32].

### Relevant concomitant care permitted or prohibited during the trial {11d}

Throughout the trial, individual diabetes management plans will be guided by their treating practitioner. Our aim is to test Freestyle Libre 2 technology in “real world settings” according to local practices in different locations. Therefore, we will not have restrictive protocols for treatment changes or insulin titration algorithms, as this would make the general applicability of our data uncertain. Participants in both groups will continue standard self-management and clinical care for their type 2 diabetes and will be instructed to contact their primary care provider for all treatment decisions. In the instance that medication changes or clinically relevant information regarding best practice for the participant’s diabetes management is raised, the treating team will liaise with the local study team including the local principal investigator to make any necessary changes, as required.

### Provisions for post-trial care {30}

Not applicable in this clinical trial. At the end of the 6-month period, participants randomised to SMBG will be offered a 6-month supply of Freestyle Libre 2 sensors and/or readers as good will for participation in the trial.

### Outcomes {12}

#### Primary outcome

The primary outcome is HbA1c. HbA1c measured by point-of-care testing device are deemed insufficient for the primary outcome. Laboratory measurements of HbA1c will be performed using the standardisation of HbA1c measurements recommended by the Royal College of Pathologists Australasia and the Australasian Association of Clinical Biochemists [33]. Pathology will be processed in line with local requirements.

#### Secondary outcomes

1. Core CGM-derived endpoints [34]:
  - Percentage (%) of time in range (target) for BGL (3.9–10 mmol/L)
  - Percentage (%) of time below range for BGL, defined as <3.9 mmol/L
  - Percentage (%) of time below range for BGL, defined as <3.0 mmol/L
  - Percentage (%) of time above range for BGL, defined by >10.0 mmol/L
  - Percentage (%) of time above range for BGL, defined by >13.9 mmol/L
  - Percentage (%) of time in tight range for BGL (3.9–7.8 mmol/L)
  - Mean and standard deviation (SD) of sensor glucose (mg/dL)
2. Safety outcomes: severe hypoglycaemia [9], defined as a severe event characterised by altered mental and/or physical status requiring third-party assistance [9], ambulance call out or hospital presentation between baseline and 6 months
3. Health economic outcomes and evaluation:

Health economics outcomes include health care costs, health-related quality of life (HRQoL) and quality-adjusted life years (QALYs). Health care costs will be estimated based on health service resource utilisation. A questionnaire with a 3-month recall period will be used to capture diabetes-related costs associated with hospitalisations. Costs associated with primary and specialist care will be estimated based on claim data in the Medicare Benefits Schedule (MBS) [35] and Pharmaceutical Benefits Scheme (PBS) [36] records. All costs will be expressed in Australian dollars (AU$).

HRQoL will be measured using the EQ-5D-5L questionnaire. The participants’ response to this questionnaire can be converted into a single value called “health utility” using the value set for the Australian population [37]. We will also report on the levels of the five dimensions. The health utility will used to calculate QALYs, a generic measure of disease burden including both the quality and quantity of a participant’s life. The EQ-5D-5L is more sensitive than the EQ-5D-3L (i.e. EQ-5D 3 levels), which has been shown to be sensitive in a previous SMBG study by our team [38].

For the economic evaluation, a trial-based cost-utility analysis will be conducted by estimating the incremental cost per QALY gained (i.e. the incremental cost-effectiveness ratio, ICER) associated with CGM compared to SMBG over the 6-month period of the trial. The total costs for each treatment arm will be based on health care costs and costs associated with blood glucose monitoring. The ICER over a lifetime horizon will also be estimated by using the UKPDS Outcomes Model [39] to simulate the impact of CGM and SMBG on the occurrence of complications and death and the associated health care costs and health utility values. Probabilistic sensitivity analysis will be conducted to capture the uncertainties in the estimates of incremental costs, incremental QALYs and ICERs.

### Exploratory outcomes

Qualitative and quantitative information will be collected from all participants including using the modified glucose monitoring satisfaction survey (mGMSS) [40] and the cultural wellbeing survey. The mGMSS was adapted in consultation and collaboration with Aboriginal and Torres Strait Islander researchers, healthcare providers and community members for this trial. Surveys will be provided to the study participants to self-complete or be completed with assistance by a study staff member.

CGM-related data will be analysed in line with the latest reporting outcomes for CGM sensors [34].

Qualitative data will be collected by phone or in-person interviews at the 6-month visit. This data will provide a nuanced understanding of participant experiences of diabetes care, technology and research. The interviews will be conducted by study coordinators known to the participants and with the consent of participants will be audio-recorded. Participants will be recruited until data saturation is achieved.

### Participant timeline {13}

The trial consists of six visits over 6 months for all participants. The study visits are shown in Fig. 1. The study visit schedule including key procedures undertaken at each visit is shown in Table 1.

**Table 1:** Trial schedule

Visit	Visit 1: screening	Visit 2: eligibility confirmation	Visit 3: randomisation	Visit 4: 3-month visit	Visit 5: blinded sensor visit	Visit 6: 6-month visit
**Timing of visit (day)**	− 30	− 14	0	90	165	180
**Visit window (days)**		− 30		± 30	± 30	± 30
Informed consent video and signed consent form	X					
Inclusion/exclusion criteria	X	X				
Medical history (including updates to medical history)	X					
Medications (diabetes/other or changes to medications)	X	X	X	X		X
Blood* and urine samples#	X			X		X
Height	X					
Weight	X			X		X
Blood pressure (systolic, diastolic blood pressure and heart rate)	X			X		X
Adverse events	X	X	X	X	X	X
Blinded sensor insertion		X			X	
Blinded sensor removal			X			X
Download blinded sensor data and view AGP report			X			X
Questionnaires			X	X		X
Diabetes education/reviews			X	X	X	X
For CGM group only: provide sensors for next visit			X	X	X	
For CGM group only: download Freestyle Libre 2 FlashGM data (sensors/readers) and view AGP report			X	X	X	X
FOR SMBG group: review SMBG data (if available)			X	X	X	X
Offer CGM to participants in SMBG arm						X
Voucher and trial interview						X

Laboratory pathology for blood and urine tests: biochemistry (bloods approximately 15–20 mL) analysed for HbA1c, blood glucose, albumin, creatinine, estimated glomerular filtration rate, full blood count, urea electrolytes and creatinine, liver function test, lipid profile urine sample analysed for urine albumin:creatinine ratio

At visit 1, trained study staff discuss the study procedures, outline the various requirements, present the informed consent video and explain the consent form to the participant. Following written informed consent, the participant is screened against the inclusion and exclusion criteria, followed by detailed documentation of the participants’ medical history including duration of diabetes and medications. Demographics, including age, remoteness, education level and smoking status, are recorded alongside weight, height and blood pressure. Blood and urine samples are taken to measure HbA1c (primary outcome), and lipid profile, full blood examination (FBE), urea electrolytes and creatinine (UEC), liver function test (LFT) and urine albumin to creatinine ratio. HbA1c results within 2 weeks prior to screening may also be used at visit 1.

At visit 2 (baseline), study staff member confirms eligibility of the inclusion criteria of a laboratory-based HbA1c ≥ 7.5% indicated by the blood sample taken at visit 1. Upon confirmation of eligibility, a Freestyle LibrePro iQ blinded sensor is inserted on the participant’s upper arm for 2 weeks by a study staff member.

At visit 3, the Freestyle LibrePro iQ blinded sensor is removed, data is uploaded and reviewed by a relevant health professional or trained study staff member and diabetes education is provided. Study questionnaires are administered, including the EQ-5D-5L. Participants are randomised to receive Freestyle Libre 2 CGM (intervention) or standard care (SMBG). Participants randomised to the intervention are shown how to insert and remove the sensor according to manufacturer instructions and how to use the handheld reader or mobile application to obtain glucose readings. Participants will be encouraged to scan at least 10 times per day. Participants in the intervention arm will be provided with sensors for 3 months.

At visit 4 (3 months), participants are reviewed by a study staff member and questionnaires (EQ-5D-5L and mGMSS) are administered. Participants randomised to the intervention will have their data reviewed by a study staff member and provided with 3 additional months of sensors. Weight and blood pressure are taken. A venous blood sample is taken to measure the primary outcome, HbA1c, including urine sample.

At visit 5, all participants will have a Freestyle LibrePro iQ blinded sensor inserted on the upper arm for 2 weeks by a study staff member.

At visit 6 (6 months), the Freestyle LibrePro iQ blinded sensor is scanned, uploaded, reviewed and removed by a relevant health professional or trained study staff member and diabetes education is provided. Study questionnaires are administered, including the EQ-5D-5L. Participants randomised to Freestyle Libre 2 intervention are reviewed and data are downloaded. Participants randomised to SMBG are reviewed by a study staff member and offered 6-month supply of Freestyle Libre 2 sensors to ensure equity between the two arms of the study. A final venous blood test is undertaken to collect the primary outcome of HbA1c at 6 months, including urine sample. All participants are offered a final trial interview with a study staff member to discuss their experience of participating in the trial. If the participant is willing to have their interview audio-recorded, the recording is stored for later analysis. Adverse events are collected at each visit.

### Sample size {14}

A sample size of 105 completed participants per arm is required for 80% power to demonstrate that CGM is superior to SMBG with a two-sided 5% significance level. This sample size is based on the following assumptions: clinically meaningful absolute treatment difference in mean change from baseline to 6 months in HbA1c of 0.5% in favour of CGM (i.e. reduction), standard deviation (SD) of 1.6% [41] equal in each arm and at each time point (baseline, 3 and 6 months), and correlation between all three measurements of 0.60 (i.e. compound symmetry variance–covariance matrix). This sample size is based on the following statistical model: constrained longitudinal data analysis (cLDA) with the response consisting of all HbA1c measurements (baseline, 3 and 6 months) and the model including factors representing treatment, time (categorical) and treatment-by-time interaction, with the restriction of a common baseline mean across treatment groups. The absolute treatment difference in mean change from baseline to 6 months is estimated and tested (*t*-test) from this model. This sample size calculation was programmed using SAS software, Version 9.4 of the SAS System for Windows and validated in Stata SE, version 14.1 (STATACorp. 2015) using both sampsi (with ANCOVA method) and power twomeans commands (with adjusted SD of 1.28%). Assuming a drop-out of approximately 40% at 6 months, a total sample size of 350 patients (*n* = 175 in each arm) will be required.

### Recruitment {15}

The first study site was activated on 6th May 2021, the first participant was recruited on 22nd June 2021 and the first participant was randomised on 13th July 2021. Each participating site will aim to recruit a minimum of 20 participants, up to a total of 350 participants for the study. A withdrawal of consent form is available for participants who choose to withdraw from the study. Data already collected will be retained and will form part of the data analysis and results. This will be explained to participants during the consent process.

### Assignment of interventions: allocation

#### Sequence generation {16a}

Stratified block permuted 1:1 randomisation will be used, with study centre as a stratification factor, to ensure balance in treatment allocation within each centre.

#### Concealment mechanism {16b}

Participants will be assigned to either the intervention or comparator group via an electronic randomisation module in REDCap hosted by the University of Melbourne (Australia). The randomisation allocation table was generated by an independent (non-study) statistician and is concealed from all staff for the duration of the study.

#### Implementation {16c}

Participants will be enrolled by their local study staff member responsible for the trial at their local health service. At visit 3, participants will be assigned to the intervention (Freestyle Libre 2) or standard care (SMBG) using a web-based randomisation system REDCap.

### Assignment of interventions: blinding

#### Who will be blinded {17a}

This is a non-masked trial as both investigators and participants are aware of the intervention (Freestyle Libre 2). Study coordinators are unblinded to treatment allocations during the trial. Statisticians are blinded until the database is cleaned and locked for analysis.

#### Procedure for unblinding if needed {17b}

The design is open label so unblinding of the investigator or the participant is not applicable.

### Data collection and management

#### Plans for assessment and collection of outcomes {18a}

##### Laboratory analysis

The primary outcome, HbA1c, will be measured from blood samples drawn at three time points: screening, 3-month and 6-month visits. At these visits, venous blood and urine samples are collected from the participant at the trial site or local pathology service, then processed through an accredited laboratory. No point of care HbA1c testing will be used for the outcomes of the trial.

#### Plans to promote participant retention and complete follow-up {18b}

Methods to promote participant retention include recruiting participants from local communities where the trial is conducted, engaging local staff to conduct the study such that culturally appropriate and safe care is maintained, access to Freestyle Libre 2 sensors either during the trial or upon completion (if randomised to standard care) and a study t-shirt and a $50 voucher upon completion of the trial. Participants who discontinue or deviate from the intervention protocol will have their data included in the study and final analysis.

#### Data management {19}

Case report forms (CRFs) are designed, constructed and maintained by study staff and hosted on a central online REDCap database hosted by the University of Melbourne. Each participating site is assigned to a unique Data Access Group that (a) ensures authorised study staff only have access to records for participants enrolled at their site and (b) randomises via the REDCap randomisation module.

Authorised study staff are granted a password-protected secure account with 2-point verification to access the REDCap portal from an internet browser. The database has facilities to transmit secure data queries and requests for missing data (directly or via email notifications) to authorised users, as well as data interrogation and cross-checking algorithms to minimise incorrect data entries and maximise data capture and completeness. Paper CRFs are available to sites as a tool to facilitate data collection and must be kept in locked cabinets/offices in accordance with ICH-GCP guidelines and local regulations.

Study data will undergo source-data verification during routine compliance monitoring. All study data must be retained by each site for at least 15 years following the completion of the trial. For participants who provided consent to data linkage (as specified on the PICF and distinct from their consent to enrolment in the trial), a minimal set of identifiable data will be provided to the Australian Institute of Health and Welfare.

#### Confidentiality {27}

All trial-related information will be stored securely. Participant information will be stored in a locked area accessible only to the research team. All paper CRFs will be identified by a unique study code to maintain participant confidentiality. All data will be entered by study staff. Paper source note data will be transposed and entered into a secure electronic database (REDCap).

The Australian Institute of Health and Welfare will be provided with specific identifiable information required for linkage for the health economic analysis of the study. All data linkage will be performed with and under the guidance of the Australian Institute of Health and Welfare. A confidentiality agreement will be signed by both the Australian Institute of Health and Welfare and the University of Melbourne (Australia) to ensure both parties adhere to confidentiality requirements for data linkage.

All local databases are secured with password-protected access systems. Participants’ study information will not be released outside of the study protocol without the written permission of the participant, except as necessary for monitoring by the Trial Coordinating Centre (The University of Melbourne, Australia), HRECs or other regulatory bodies. All study-related matters remain confidential. Study staff working on the trial will be required to maintain confidentiality and, where necessary, sign confidentiality agreements prior to engaging with the study. This includes individuals and parties external to the study team, who are named on committees or may be affiliated with the trial. Any person/s engaging with the study will be briefed on the importance of confidentiality, in line with ICH-GCP requirements.

#### Plans for collection, laboratory evaluation and storage of biological specimens for genetic or molecular analysis in this trial/future use {33}

No biological specimens will be stored for this trial. Biological specimens will only be used for the purposes of routine pathology (blood and urine samples) required for the primary outcome, HbA1c.

### Statistical methods

#### Statistical methods for primary and secondary outcomes {20a}

A detailed statistical analysis plan will be finalised and uploaded onto the University of Melbourne institutional repository (figshare.unimelb.edu.au) prior to database lock [42]. This plan will document the precise analysis methods to obtain the treatment effects of interest as well as any sensitivity and supplementary analyses. The primary outcome HbA1c will be analysed using a likelihood-based longitudinal model (i.e. cLDA) [43] with response consisting of all values (baseline, 3 and 6 months) and the model including factors representing treatment group, time point and treatment group by time point interaction. The stratification factor study centre will be included in the model as a main effect. The model will assume a common mean across treatment groups at baseline and an unstructured variance–covariance matrix to account for the repeated outcome data collected on each participant. The absolute treatment difference in mean change from baseline to 6 months will be estimated and a two-sided 95% confidence interval and *p* value will be obtained. The model will include all participants who have at least one HbA1c measurement. Continuous secondary outcomes will use a model similar to that for the primary outcome, after appropriate transformation of the outcome before fitting the model only if required.

##### Health economic analyses

The economic evaluation will involve a cost-utility analysis. This will be based on the comparison between the intervention (CGM) and standard care (SMBG) in terms of costs and QALYs. Data on costs and outcomes will be collected prospectively.

Cost incurred by each participant in each treatment arm will be calculated based on cost of intervention and the downstream costs of healthcare services and hospitalisations. For the intervention cost, we will quantify the resources required for the Freestyle Libre 2 system including the costs associated with training and consumables and will collect information on the average time taken to deliver the intervention relative to standard care.

For the downstream costs, we will ask health professionals to capture the average time each person spends with a participant explaining the use of the intervention. Healthcare professionals’ years of experience and expertise levels will be captured. In addition, wages for the different HCPs will be sought to determine an estimated delivery of intervention cost. Healthcare resource use will be quantified by tracking participant’s use of items in the Medicare Benefits Scheme (MBS) and Pharmaceutical Benefits Scheme (PBS) databases from Services Australia. Participants will need to provide consents to linkage of their trial data with MBS and PBS records.

Hospital costs will be measured using a health services and hospital use questionnaire. We will use data obtained during the study to estimate the incremental cost associated with delivering the intervention if rolled out at a national level.

For outcomes, we will use an established algorithm to convert responses to the EQ-5D-5L to preference-based measures of health-related quality of life (HRQoL) and from this calculate health gain associated with intervention in terms of incremental QALYs. QALYs will consider both changes in HRQoL and impact on life expectancy from reductions in HbA1c. Cost-utility will be assessed by estimating the incremental cost per QALY gained. We will follow the approach proposed by Sterne et al. to analyse missing data and perform multiple imputation, when appropriate, to replace the missing risk factors with the estimates. For the trial-based cost-utility analysis, we will use bootstrap inference to capture the uncertainty surrounding costs, QALYs and incremental cost-effectiveness ratio (ICER) and to construct a cost-effectiveness acceptability curve representing the probability of Freestyle Libre 2 being cost-effective at different willingness-to-pay thresholds [44]. We will also conduct sensitivity analysis to determine impact of changes in the estimates on the ICER, based on which identify future studies needed to enhance the accuracy of the estimated ICER whether Freestyle Libre 2 represents value for money. For the model-based cost-utility analysis, we will extrapolate the effects of Freestyle Libre 2 and SMBG on HbA1c beyond the trial period and use the UKPDS Outcomes Model to simulate the impact of these interventions on changes in risk factors and occurrence of complications and death, based on which the ICER over a lifetime horizon will be estimated.

#### Interim analyses {21b}

No interim analyses will be conducted for the trial.

#### Methods for additional analyses (e.g. subgroup analyses) {20b}

All methods of analysis including additional analyses will be outlined in the statistical analysis plan. Exploratory subgroup analysis will be performed for the primary outcome at 6 months for the intention to treat population. The subgroup analysis listed below will be performed irrespective of whether the primary objective of the study (HbA1c) at 6-month follow-up is achieved or not. The subgroups are duration of diabetes, baseline HbA1c, diabetes medication type, location (remoteness) and presence of diabetes complications, in particular diabetic kidney disease.

#### Methods in analysis to handle protocol non-adherence and any statistical methods to handle missing data {20c}

The primary outcome HbA1c will be analysed using a likelihood-based longitudinal model. The primary strategy to handle missing data will utilise a likelihood-based approach. This provides valid inference in the presence of missing data if the missing data mechanism is ignorable (i.e. missing completely at random or missing at random). The primary model will include all participants who have at least one HbA1c measurement irrespective of adherence.

#### Plans to give access to the full protocol, participant-level data and statistical code {31c}

The full protocol will be available upon request. Requests for any participant-level datasets and statistical code may be available upon request and with the consent of the trial sites.

### Oversight and monitoring

#### Composition of the coordinating centre and trial steering committee {5d}

##### Trial Management Group (Study Leadership Team)

The Study Leadership Team oversees all aspects of the trial and its operation, including the day-to-day operation of the trial. The team comprises of the lead investigator (EIE), Aboriginal researchers and community members (RK and TH), trial manager (MH), trial data manager (CS) and lead study coordinator (BM). The Trial Management Group meets weekly. Aboriginal and Torres Strait Islander leadership is driven by RK and TH. Day-to-day management of the trial is driven by MH. Trial oversight is driven by lead investigator, EIE.

##### Trial Chief Investigators Committee

The Trial Chief Investigators Committee are all the sites, chief investigators, associate investigators and collaborators, principal investigators and study staff who work directly on the trial. Meetings will be conducted face to face and/or by teleconference every 6 months after commencement of the trial or as needed. Membership includes all chief investigators, associate investigators, collaborators, study staff, sites and principal investigators.

##### Aboriginal and Torres Strait Islander Community Advisory Committee

The Aboriginal and Torres Strait Islander Community Advisory Committee oversees Aboriginal and Torres Strait Islander data governance and sovereignty, to minimise the risks of research for Aboriginal and Torres Strait Islander research participants and communities through the adoption of culturally safe research practices. The Aboriginal and Torres Strait Islander Community Advisory Committee is led by TH and supported by RK. The committee consists of Aboriginal and Torres Strait Islander community members (past participants) invited from various communities involved in the FlashGM Study. The committee meet quarterly to discuss community perspectives on the trial conduct, including strategies for wider community engagement and dissemination of findings.

##### Study Coordinator Committee

The Study Coordinator Committee meets monthly (or where appropriate, quarterly) to discuss trial conduct across sites, exchange learnings, explore developments in diabetes management and serve as a networking forum and professional development exchanges to increase research capability and capacity at individual, organisation and community level. The committee is led by the lead study coordinator (BM) and comprises of all study coordinators working on the trial, including supporting staff involved in the trial.

##### Data Quality Committee

The Data Quality Committee reports to the Leadership Team regarding data collection, completeness and database management for the trial. Meetings will be conducted face to face and/or by teleconference every 6 months after commencement of the trial or as needed.

Membership includes trial biostatisticians (SB and DNK), trial manager (MH) and data manager (CS).

##### Writing Committee

The Writing Committee is responsible for planning, writing and submitting for publication, all reports of the trial methods, progress and results. Membership: FlashGM Leadership with comments and reviews by CIs, AIs and collaborators.

#### Composition of the data monitoring committee, its role and reporting structure {21a}

An independent Data Safety Monitoring Board (DSMB) comprising of a clinical endocrinologist chair, general practitioner with experience in Aboriginal health, senior expert trial manager and senior statistician has been appointed. The DSMB are notified of all serious adverse events, including device-related adverse events during the study and independently review the conduct and safety of the trial for participants. The DSMB meet 6 monthly (or where necessary, 3 monthly) to review open reports (masked to treatment allocation) and closed reports (unmasked and not available to members who are also involved in the study). Reports outline serious adverse events, any unanticipated adverse events, recruitment and impediments to study progress. The DSMB report to the Trial Management Team regarding safety concerns and recommendations for study continuation (including early termination) of the trial.

#### Adverse event reporting and harms {22}

The following definitions will apply to the FlashGM Study trial, as per the latest guidance provided by the NHMRC. The current definitions from the NHMRC Guidance: Safety monitoring and reporting in clinical trials involving therapeutic goods (Nov 2016) will be used.

Primary, secondary and other outcomes and all data collected in the CRF are not to be reported as adverse events (AEs), serious adverse events (SAEs) or suspected unexpected serious adverse reactions (SUSARs). The data safety monitoring committee will be responsible for assessing the severity, relatedness and outcomes of the AEs. SAEs and SUSARs are to be reported to the Trial Coordinating Centre (The University of Melbourne), within 24 h of the site investigators becoming aware of the event, using the reporting forms provided to the sites (both electronic and paper versions).

Serious adverse events of interest include (i) hypoglycaemic episodes requiring third-party assistance, including events of severe hypoglycaemia, defined as a severe event characterised by altered mental and/or physical status requiring third-party assistance, and (ii) hospital admission, defined by an overnight admission to hospital (> 24 h). All events are to be documented in the adverse events CRF and graded on a scale of intensity (grade 1–5, with 1 as mild to 5 as death related to AE), attribution (grade 0 as not related to grade 4 definitely related) and outcome (grade 0 as unknown/lost to follow-up to grade 4 as fatal).

#### Frequency and plans for auditing trial conduct {23}

##### Study monitoring

Monitoring will be performed in accordance with the ICH-GCP guidelines (Trial Monitoring Plan available upon request). All participating sites will undergo routine compliance monitoring, by way of centralised, remote and on-site monitoring. Following on-site monitoring, an audit may be required and will be at the discretion of the Leadership Team. The Data Quality Committee will conduct regular, blinded data quality and monitoring reviews and report to the Trial Leadership Team.

#### Plans for communicating important protocol amendments to relevant parties (e.g. trial participants, ethical committees) {25}

Any modifications to the protocol which may affect the conduct of the study, potential benefit of the patient or may affect patient safety, including changes of study objectives, study design, patient population, sample sizes, study procedures or significant administrative aspects, will require a formal amendment to the protocol. Such amendments will only be made by the Leadership Team and will be submitted for approval by the lead HREC for all sites. HREC approval of any amendments will be forwarded to all sites. All sites must obtain HREC approval for protocol amendments prior to implementation in accordance with local regulations.

### Dissemination plans {31a}

Community feedback will be sought regarding the best dissemination methods of the research findings for each community. Alongside community consultations and feedback, the research will be translated into a plain language document and/or media highlighting the key study findings. The results of the study will be written and published in an open access journal. The published research articles, including any media, will be circulated to participants and clinical trial sites. Community sessions will be held to give feedback on the findings to participants, study staff and community members. Elements of this study (including results) will also be used by researchers on this study to obtain higher degrees (PhD, Masters).

## Discussion

Relatively few clinical trials have been undertaken in Aboriginal and Torres Strait Islander primary health services to this scale [45]. To the best of our knowledge, this is Australia’s first large, national, multi-centre randomised controlled trial for Aboriginal and Torres Strait Islander peoples with type 2 diabetes using CGM. Aboriginal and Torres Strait Islander people are underrepresented in clinical trials globally [46]. This is largely attributed to the ongoing effects of colonisation and the history of mistrust, racism and subsequent fear of non-Aboriginal and Torres Strait Islander healthcare system, professionals and research institutions [46]. Yet the evidence generated in primary care settings may be more translatable across diverse populations, contexts and priority conditions than evidence from tertiary settings. Primary care services, such as Aboriginal Medical Services (AMSs) and Aboriginal Community Controlled Health Organisations (ACCHOs), aim to provide holistic health and well-being services for Aboriginal and Torres Strait Islander communities [47]. Clinical trial sites for this study are predominantly based in rural, regional and remote AMSs and ACCHOs which builds upon a strengths-based participatory model of involving local people, networks and healthcare providers to foster trust, relationships and open communication.

### Extenuating circumstances: COVID-19

The trial commenced at the onset of the COVID-19 pandemic. This resulted in a delay in initiation and recruitment of the study due to severe COVID-19 lockdowns, furloughing of staff members and isolation measures to protect Aboriginal and Torres Strait Islander communities [48]. There was a significant reduction in clinical research trials which were allowed to continue during this time as priorities shifted to protect vulnerable communities. This resulted in a series of lockdowns with differences at regional and state level in Australia. Subsequently, due to these differences, recruitment was staggered and resulted in our first participant being screened in June 2021. Ensuring local priorities were respected, the study continued cautiously as sites emerged from lockdown. As the COVID-19 vaccination became widely available, efforts by local health services and sites were focused on ensuring Aboriginal and Torres Strait Islander communities were protected and vaccinated [49]. The COVID-19 pandemic significantly impacted our ability to recruit and engage with sites face-to-face for 2 years, between 2020 and the end of 2021. Despite this, we were able to develop the informed consent video for the study, conduct remote meetings with teams, develop the study materials and publish the Culturally Adaptive Governance Framework [50].

In addition to the COVID-19 pandemic and severe lockdowns, between 2021 and present, clinical trial sites have been affected by natural disasters such as flooding, technological challenges including outages and cyberattacks, and an ongoing global shortage of GLP-1 medication [32]. The latter has significantly impacted trial sites to screen and recruit participants for the study, as one of the inclusion criteria requires a participant to be medicated with the same type of injectable therapies with/or without oral hypoglycaemic agents, for at least 6 weeks prior to screening. Due to the popularity of GLP-1 medications, this global shortage has impacted a significant number of potential participants who would otherwise be eligible for the study. Additional challenges include workforce turnover and staff leave, including recognition that clinical trial staff at sites are primarily health professionals undertaking research as an additional duty [51]. Additionally, respect for culture and family priorities such as Sorry Business, change of location due to family commitments or work demands, may mean that some study visits may be missed. The study aims to undertake a pragmatic approach to conducting research with respect for participants and local community priorities.

The trial is ongoing despite these challenges, which is a testament to the dedication of communities, workforce and participants involved in this study.

## Trial status

The trial is conducted using Protocol v1.4, 1st June 2023. Recruitment commenced on 22nd June 2021. The trial is currently recruiting, with anticipated completion by December 2025.

## Data Availability

The Leadership Team and the Aboriginal and Torres Strait Islander Community Advisory Committee will oversee data sharing. Site principal investigators will have direct access to their own site’s data sets. Applications to access data for secondary studies or other research purposes can be forwarded to the Leadership Team and the Aboriginal and Torres Strait Islander Community Advisory Committee for consideration. The final trial dataset will be available to the University of Melbourne for analysis. Each site will have access to and own the data from their own community.

## Abbreviations

ACCHO: Aboriginal Community Controlled Health Organisation
AHMRC: Aboriginal Health and Medical Research Council
AGP: Ambulatory glucose profile
AMS: Aboriginal Medical Service
ANCOVA: Analysis of covariance
BGL: Blood glucose level
cLDA: Constrained longitudinal data analysis
CGM: Continuous glucose monitoring
CRF: Case report form
FlashGM: Flash Glucose Monitoring
GLP-1: Glucagon-like peptide 1
HbA1c: Glycated haemoglobin
HCP: Healthcare practitioner
HRQoL: Health-related quality of life
ICER: Incremental cost-effectiveness ratio
MBS: Medicare Benefits Schedule
mGMSS: Modified glucose monitoring satisfaction survey
NACCHO: National Aboriginal Community Controlled Health Organisation
PBS: Pharmaceutical Benefits Scheme
PICF: Participant informed consent form
QALYs: Quality-adjusted life years
REDCap: Research Electronic Data Capture
SD: Standard deviation
SMBG: Self-monitoring blood glucose
