# Correction: Can flash glucose monitoring improve glucose management for Aboriginal and Torres Strait Islander peoples with type 2 diabetes? A protocol for a randomised controlled trial

**DOI:** 10.1186/s13063-024-08363-8

**Published:** 2024-08-05

**Authors:** Mariam Hachem, Tracey Hearn, Ray Kelly, Audrey Eer, Belinda Moore, Christine Sommerville, Sharon Atkinson-Briggs, Stephen Twigg, Meagan Freund, David O’Neal, David Story, Alex Brown, Anna McLean, Ashim Sinha, John Furler, Richard O’Brien, An Tran-Duy, Philip Clarke, Sabine Braat, Digsu N. Koye, Sandra Eades, Luke Burchill, Elif Ekinci

**Affiliations:** 1 Department of Medicine, Austin Health, Faculty of Medicine, Dentistry and Health Sciences, University of Melbourne, Melbourne, Victoria Australia; 2 Endocrinology, Austin Health, Melbourne, Victoria Australia; 3 Australian Centre for Accelerating Diabetes Innovations (ACADI), Melbourne Medical School, Faculty of Medicine, Dentistry and Health Sciences, University of Melbourne, Melbourne, Victoria Australia; 4 Medical Clinic, Rumbalara Aboriginal Cooperative, Mooroopna, Victoria Australia; 5 Implementation Science Research Group, University of Melbourne, Melbourne, Victoria Australia; 6 School of Medicine and Public Health, College of Health, Medicine and Wellbeing, Health Behaviour Research Collaborative, University of Newcastle, Newcastle, New South Wales Australia; 7 Department of Medicine, Faculty of Medicine, Dentistry and Health Sciences, University of Melbourne, Melbourne, Victoria Australia; 8 Endocrinology, St Vincent’s Hospital Melbourne, Melbourne, Victoria Australia; 9 Critical Care, Faculty of Medicine, Dentistry and Health Sciences, University of Melbourne, Melbourne, Victoria Australia; 10 Indigenous Genomics, Telethon Kids Institute, Adelaide, South Australia Australia; 11 Indigenous Genomics, The Australian National University, Canberra, Australian Capital Territory Australia; 12 Department of Endocrinology and Diabetes, Cairns Hospital, Cairns, Queensland Australia; 13 Department of General Practice, University of Melbourne, Melbourne, Victoria Australia; 14 Centre for Health Policy, Melbourne School of Population and Global Health, University of Melbourne, MelbourneMelbourne, Victoria Australia; 15 Methods and Implementation Support for Clinical and Health research (MISCH) Hub, Faculty of Medicine, Dentistry and Health Sciences, University of Melbourne, Melbourne, Victoria Australia; 16 Health Economics Research Centre, University of Oxford, Oxford, United Kingdom; 17 Centre for Epidemiology and Biostatistics Melbourne School of Population and Global HealthFaculty of Medicine, Dentistry and Health Sciences, University of Melbourne, Melbourne, Victoria Australia; 18 Cardiovascular Medicine, Rochester, Minnesota United States of America

**Correction: Trials 25, 493 (2024)**

**https://doi.org/10.1186/s13063-024-08267-7**

Following the publication of the original article [1], we were notified that the name of the 17^th^ author was missing a hyphen.

Originally published name: An Tran Duy

Corrected name: An Tran-Duy

The original article has been corrected.
